# Reversing the Photovoltage of a Photoacid in Aqueous Glycerol and Ethylene Glycol Solutions

**DOI:** 10.1021/acsomega.6c00091

**Published:** 2026-01-29

**Authors:** Lars Egil Helseth

**Affiliations:** Department of Physics and Technology, 87344 University of Bergen, Allegaten 55, 5020 Bergen,Bergen 5020, Norway

## Abstract

Pyranine is a photoacid, which upon illumination in water, deprotonates. It has been well studied and can therefore be used as a model system for developing sensors and energy-harvesting devices based on photoacids. Under normal circumstances, the release of protons results in an open-circuit voltage of a fixed polarity. Here, it is demonstrated that the polarity of the photovoltage in glycerol and ethylene glycol is reversed compared to that in water. The polarity and kinetics of the photovoltage are found to depend on the ratio of glycerol or ethylene glycol to water. A simple model for the observed photovoltage kinetics is proposed, based on the assumption that both positive and negative charges are released and exhibit different diffusion constants.

## Graphical abstract

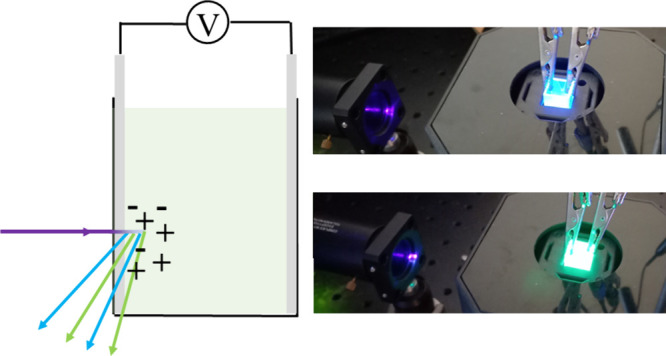

## 1 Introduction

Photoacids release protons when illuminated and are under consideration for various applications in sensing,^1–5^ controlling sol–gel transitions,^6^ light-driven ion transport in membranes,^7^ applications for conversion of light to electrical energy,^8–11^ or pn-junction for self-powered photoelectric sensors.^12^ Photovoltages of the order of 0.2 V have been achieved in the solid state by covalent linkage of a photoacid to a gold surface.^13^

Pyranine is a particularly well-characterized fluorescent photoacid, which can be used as a pH indicator in the ground state or as proton transfer probe in the excited state.^14^ As a pH sensor, it has been used for example to detect phase changes^2^ and monitor different microscale environments.^3,14,15^ Pyranine can assist the self-assembly of polyelectrolytes into colloids with pH-dependent fluorescence.^16^ It can also be used in quenching mode to detect small concentrations of for example paraquat^4^ or copper ions.^5^ Pyranine has been used as a molecular probe of different solvent environments.^17^ The excited state depends on the solvent such that an aprotic environment may give rise to a nonpolar excited state, whereas a more polar environment allows charge transfer properties.^18^ The protonation of pyranine can be controlled by its microenvironment, and this has been used to investigate the dynamics of colliding droplets of methanol and water.^19^

The light-switchable proton conductivity of pyranine has been observed in water, polymeric solutions^20^ and melting coordination polymer crystals.^21^ Impedance spectroscopy and open-circuit voltage measurements have indicated that the photoelectrochemical response of pyranine is governed by protons at the electrode–electrolyte interface.^22^

In the current work, it is demonstrated that in glycerol and ethylene glycol, the photovoltage due to pyranine is reversed as compared to water, thus suggesting that opposite charge carriers are released near the electrode. The influence of the mixing ratio on the photovoltage kinetics is investigated, and a simple model based on diffusion is proposed to explain the experimental data.

## 2 Materials and Methods

In the current study, deionized and ultrapure water was used (resistivity of 18.2 MΩcm, Millipore). The glycerol was 99% pure (Sigma-Aldrich G9012—500 mL), the methanol was ≥99.8% pure (Sigma-Aldrich 32213—1 L), and the ethylene glycol was ≥99% pure (Sigma-Aldrich 293237—1 L). The polar solvents were mixed with water in different molar ratios. 8-Hydroxypyrene-1,3,6-trisulfonic acid trisodium salt (HPTS, Sigma-Aldrich H15291G), hereafter referred to as pyranine, was mixed into the solutions.

About 0.8 mL of solution was filled in plastic cuvettes with a 5 mm path length. Upon illumination with a 5 mW 404 nm diode laser (Thorlabs), a fluorescence spectrum was emitted, as can be seen for different molar fractions of methanol and water in Figure 1a or for glycerol–water mixtures in Figure 1b. The fluorescence spectrum was collected by an optical fiber and measured using a spectrometer (Ocean Optics QE 65000).

**Figure 1:**
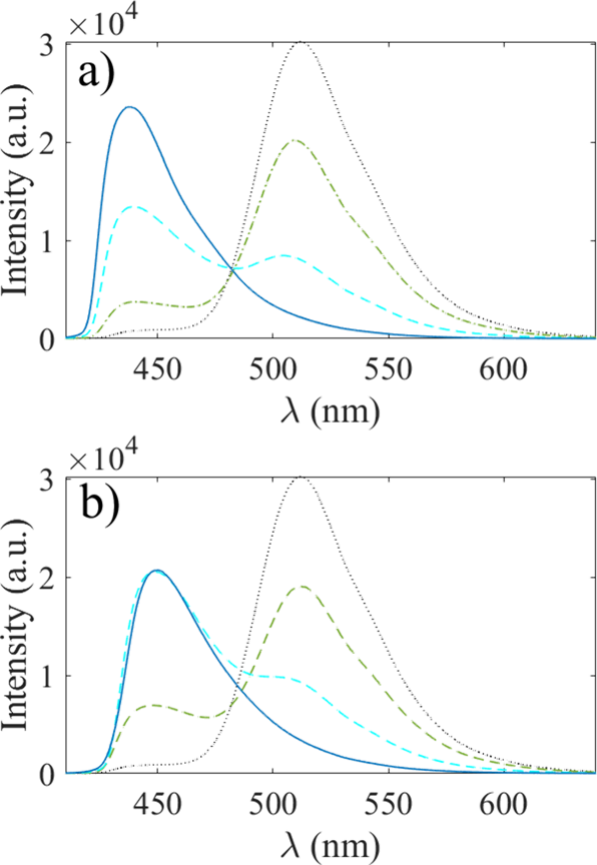
In panel (a), the fluorescence emission of 1 mM pyranine is shown for methanol molar fractions χ = 0 (black, dotted line), χ = 0.2 (green dash-dotted line), χ = 0.7 (cyan dashed line), and χ = 1 (blue line). In panel (b), the fluorescence emission of 1 mM pyranine is shown for glycerol molar fractions χ = 0 (black, dotted line), χ = 0.3 (green dash-dotted line), χ = 0.5 (cyan dashed line), and χ = 1 (blue line).

To clearly see the difference in fluorescence emission wavelength peaks between the different solvents, Figure 2a shows the normalized intensity of 1 mM pyranine in methanol (dotted violet line), ethylene glycol (brown line), glycerol (blue dashed line), and water (green line) as a function of wavelength. It is found that 1 mM pyranine gives peak fluorescence at 438 nm in methanol, 443 nm in ethylene glycol, 450 nm in glycerol, and 512 nm in water. The blue intensity peak corresponding to protonated pyranine is denoted as *I*_blue_, whereas the deprotonated green intensity peak is *I*_green_. The ratio *I*_blue_/*I*_green_ at different molar fractions is shown in Figure 2 b for glycerol (blue triangles), ethylene glycol (brown squares), and methanol (violet circles).

**Figure 2:**
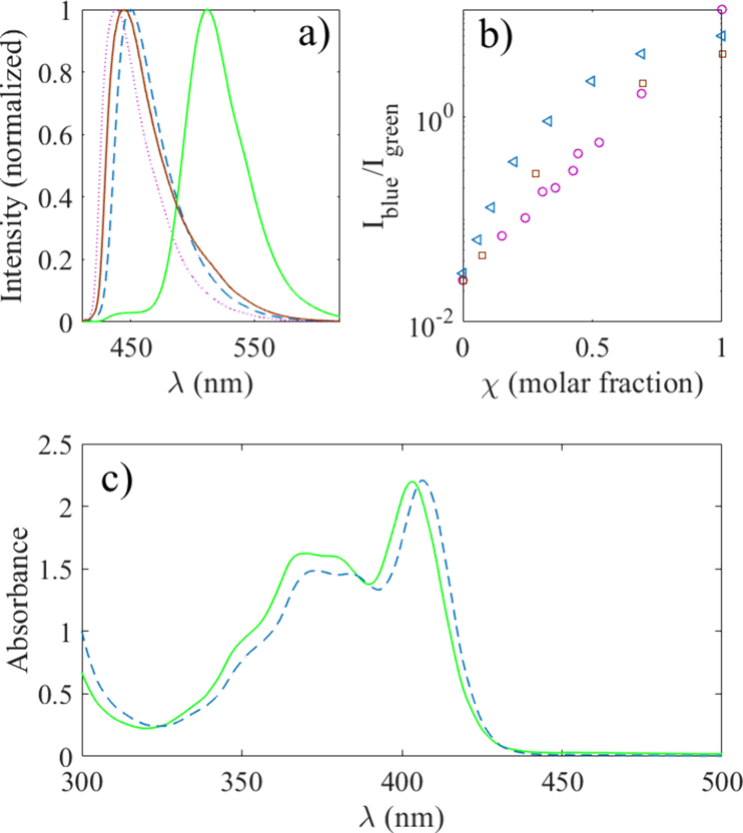
In panel (a), the normalized fluorescence emission of 1 mM pyranine in methanol (dotted violet line), ethylene glycol (brown line), glycerol (blue dashed line), and water (green line) is shown as a function of wavelength. In panel (b), the ratio between the protonated (*I*_blue_) and deprotonated (*I*_green_) intensity peaks from glycerol (blue triangles), ethylene glycol (brown squares), and methanol (violet circles) is shown as a function of solvent molar fraction. In panel (c), the UV–vis absorbance is shown as a function of wavelength for water (green line) and glycerol–water molar fraction χ = 0.6 (blue, dashed line) both containing 0.1 mM pyranine.

Examples of the absorption spectra of 1 mM pyranine are given in Figure 2c) for pure water (solid green line) and glycerol–water molar fraction χ = 0.6 (blue, dashed line). In general, the absorbance changed little between the different solvents.

The photoresponse of the different glycerol/water mixtures was investigated using the setup in Figure 3. A 5 mW 404 nm diode laser (Thorlabs) was used to provide continuous illumination of the sample. Transparent indium tin-oxide (ITO) electrodes were glued to the cuvette walls using polydimethylsiloxane (PDMS) as shown in Figure 3a). Approximately 0.8 mL of pyranine in the appropriate mixture was filled in the cuvette. The distance between the ITO electrodes was *L* = 5 mm, with an area *A* = 140 mm^2^ in contact with liquid. The samples were illuminated with the laser through one of the ITO windows, as shown in Figure 3a). The absorbance of 1 mM pyranine is 3.9 × 10^3^ m^–1^, such that 98% of the 404 nm laser light has been absorbed after 1 mm. Thus, the laser light is absorbed close to the nearest electrode. Crocodile clamps connected the ITO to a Gamry ref 600 potentiostat, which was used to measure either the open-circuit voltage or the impedance spectrum in potentiostatic mode with zero bias and sinusoidal excitations of amplitude 150 mV.

**Figure 3:**
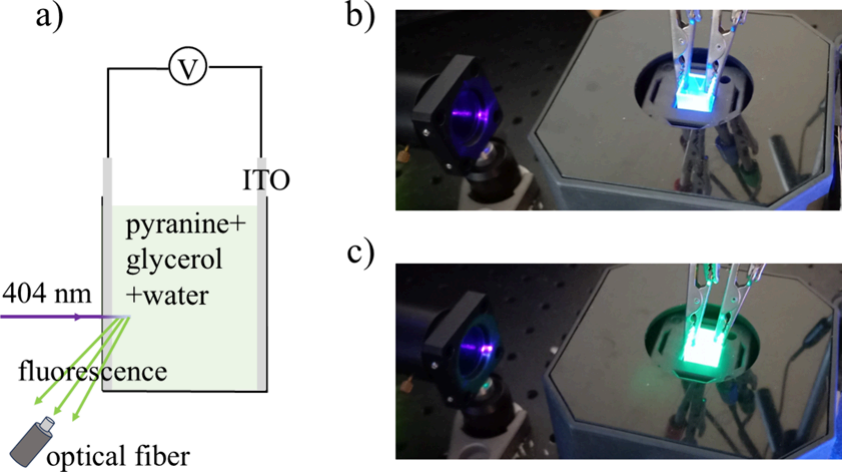
In panel (a), a schematic drawing of the setup is shown. The fluorescence is measured using an optical spectrometer connected to the optical fiber and the voltage using a potentiostat. In panels (b,c), the actual setup is shown exhibiting the fluorescence of pyranine while protonated (b) and deprotonated (c), respectively.

The setup was tested with reference solutions, such as 1 mM NaCl. The solution is first held in the dark for an hour, over which the voltage drifts less than 1 mV, as seen in Figure 4a. Also in the case of 1 mM pyranine in water, glycerol, or ethylene glycol, the drift in darkness was typically below 1 mV per hour. Upon turning on the light, a small change in voltage could be seen also in solutions that were not photoactive, probably due to thermal effects wherein the high-mobility sodium ions move away from the ITO electrode, leaving a net negative charge. An example is shown for 1 mM NaCl in Figure 4a), where the laser illumination starts at 3730 s, after which the voltage saturates at small positive value 2 mV higher than the starting point about 5000 s later. Note that the sign of the voltage is given by the manner in which the crocodile clips were attached during all the experiments, with nominal negative polarity where the laser beam enters and positive polarity at the electrode furthest away from the laser. Therefore, a negative voltage suggests accumulation of positive charge at the electrode, where the laser beam enters, and vice versa.

**Figure 4:**
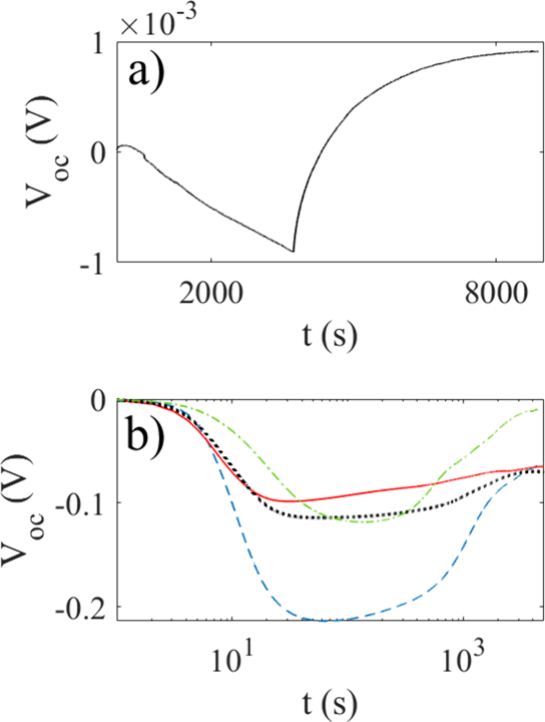
Open-circuit voltage of a 1 mM NaCl reference solution (a). The solution is first held in the dark, and the voltage drifts to −1 mV, and at 3730 s, the laser is turned on. In panel (b), the open-circuit photovoltage of 0.33 mM pyranine (green dash-dotted line, 1 mM pyranine (blue, dashed line), 3.3 mM pyranine (black dotted line), and 10 mM pyranine (red line) is shown as a function of time after the laser light was turned on at *t* = 0 s.

Figure 4b shows the measured open-circuit voltage upon illuminating 0.33 mM pyranine (green dash-dotted line, 1 mM pyranine (blue, dashed line), 3.3 mM pyranine (black dotted line), and 10 mM pyranine (red line) after the laser light was turned on at *t* = 0 s. The open-circuit voltage for all the concentrations is seen to first increase and remain at a maximum for a few hundred seconds, after which it starts to decrease. For the three largest concentrations (1, 3.3, and 10 mM), the photovoltage eventually becomes approximately −70 mV after 2 h, whereas for the smallest concentration (0.33 mM), it becomes of the order of −10 mV.

## 3 Results and Discussion

A main finding reported here is that the polar protic solvents glycerol and ethylene glycol give rise to an opposite photovoltage polarity of that observed in water. This is shown for pure glycerol (green line) and ethylene glycol (red line) in Figure 5a). Note that in both cases, the voltage grows gradually before saturating, and the process takes 30 min for ethylene glycol and almost 3 h for glycerol. In the case of water, the polarity is negative as shown in Figure 4b or the blue-dashed line in Figure 5a.

**Figure 5:**
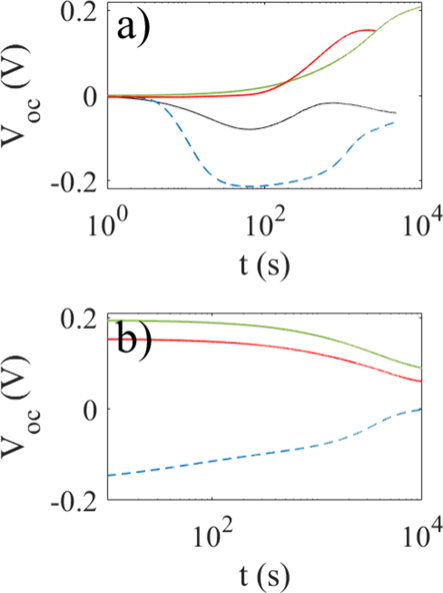
(a) The open-circuit photovoltage of a 1 mM pyranine in water (blue, dashed line), methanol (black line), ethylene glycol (red line), and glycerol (green line). Laser light illumination starts at *t* = 0 s. (b) After the laser light is removed, the photovoltage decays as shown for water (blue, dashed line), ethylene glycol (red line), and glycerol (green line).

Upon turning off the laser light, the photovoltage went to zero after about 2.8 h in the case of water as shown in Figure 5b). Note that in Figure 5b, the laser was turned off while the photovoltage was close to maximum (reached after about 100 s for 1 mM pyranine in water), but it should be mentioned that turning it off after reaching the saturation level value of about −70 mV (reached after 2 h) seen in Figure 4b also gives rise to a decay reaching zero in 2–3 h. In the case of ethylene glycol and glycerol, the decay was much slower. In fact, for glycerol, one could wait for 24 h and the photovoltage would still remain about 40–50 mV. If the decay time scales with viscosity, then one would have to wait for more than a thousand hours for the voltage to go to zero for glycerol since it takes 2–3 h for water, but an investigation into this is outside the scope of the current work.

Impedance spectra of pyranine using Nyquist plots^20,22^ or Bode plots^22^ have been useful to identify charge dynamics. Here, a comparison between water and glycerol loaded with pyranine was made in an attempt to identify the differences between the two. Figure 6a,b shows Bode plots of 1 mM pyranine in water without (black dashed line) and with (blue line) laser illumination. It is observed that the modulus of the impedance (|Z|) is hardly influenced by the illumination, whereas the phase (φ) increases significantly at frequencies below *f* = 1 Hz. At such low frequencies, the modulus |Z| is nearly inversely proportional to the frequency, and the phase is close to −90° in absence of light, thus suggesting that the system can be modeled by an equivalent series circuit consisting of a resistance (due to charge and diffusional mass transfer) and a capacitor (due to the electrochemical double layer) in parallel. Upon illumination, the phase increases to about −60° at *f* = 10 mHz while the modulus remains mainly unchanged, in qualitative agreement with Figure 4 in ref 22. This change can be attributed to a change in the diffusional mass transfer near the electrodes, although a detailed impedance model is not within the scope of the current work.

**Figure 6:**
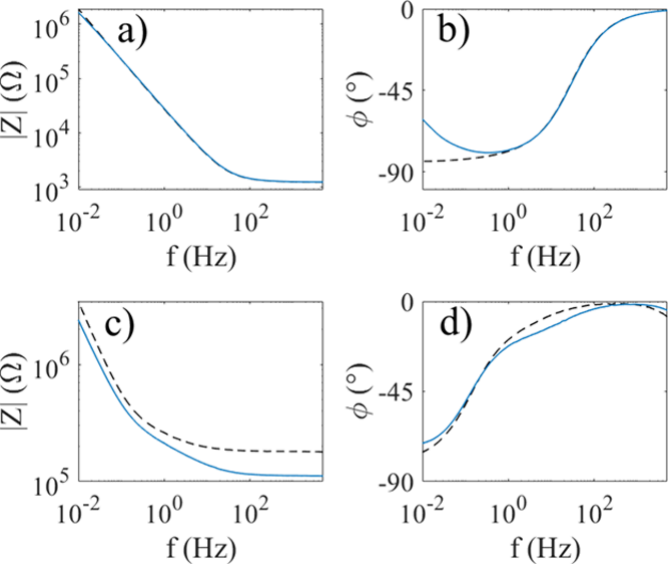
Bode plots in the frequency range 10 mHz to 5 kHz for the modulus and phase of water (a,b) and glycerol (c,d) without (black dashed line) and with (blue line) laser light.

In water, positive charges are released near the closest ITO-electrode to give rise to a negative potential, as shown schematically in Figure 7. Significant evidence points to hydrogen ions being the positive charge.^14,15,20,22^ For large pyranine concentrations (10 mM) seen in Figure 4b, most of the laser light is absorbed within 0.1 mm of the left electrode, causing a rate-limited increase in charge density and photovoltage until a maximum is reached. This maximum could be a result of overpopulation of ions at the electrode near the laser beam, and subsequent decrease in voltage might then be caused by either light saturation effects, heating, or subsequent diffusion away from this large concentration.

**Figure 7:**
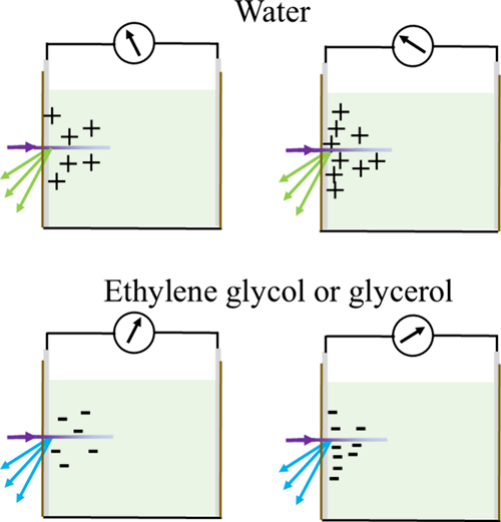
Simplified schematic drawing of the initial evolution of charge and voltage with time in water, ethylene glycol, and glycerol.

For intermediate concentrations (1 mM), most of the laser light is absorbed within 1 mm of the left electrode, and more of the ions diffuse toward the electrode from the bulk as illustrated in Figure 7. The slower buildup of charge, assisted by diffusion from the bulk toward the electrode, allows a higher charge density and therefore also photovoltage. After a few hundred seconds, the charge density at the electrode reaches a maximum, and the ions start to gradually diffuse away from the large concentration, before eventually approaching a near equilibrium situation with a photovoltage of about −70 mV. For 0.33 mM, the laser light can penetrate much further into the liquid in the cuvette, and the positive ions are fewer and will eventually diffuse into a more distributed charge density with lower photovoltage. One could argue that the increase in voltage after a few hundred seconds is due to photothermal effects, as is most likely seen for 1 mM NaCl in Figure 4a. The dye methyl red is known to have an absorbance peak in the region 400–500 nm in its acidic form, and a 1 mM solution of methyl red in water exhibited a photovoltage that gradually increased by about 4 mV in 1 h. The photothermal voltages from both 1 mM NaCl and 1 mM methyl red are much smaller and increase much slower than those for pyranine observed in Figure 4b. The voltage kinetics observed does not appear to support a hypothesis that the increase in voltage in Figure 4b is a photothermal effect. Instead, we suggest that it is caused by secondary diffusion, as described briefly above. Since both high and intermediate pyranine concentrations eventually give rise to the same photovoltage, it is likely that in this equilibrium situation, the charge distributions are comparable, as dictated by the Poisson equation and caused by diffusion of ions due to concentration changes.

Here, we selected a pyranine concentration of 1 mM for further investigation for two reasons. First, it is below the solubility limit for all of the selected liquids. Second, this concentration gives rise to a large photovoltage in water and glycerol under conditions in which the initial increase in photovoltage appears to be driven by diffusional mass transfer.

For 1 mM pyranine in glycerol, the Bode plots reveal that the modulus |Z| decreases significantly upon illumination; see Figure 6c. It is seen that at frequencies above *f* = 100 Hz, the modulus |Z| decreases from 185 to 112 kΩ upon illumination, while the phase does not change significantly. This is an indication that the ohmic serial resistance decreases. In pure water, sodium ions dissociate from the sulfonate groups of pyranine, thus increasing the ion conductivity of the glycerol solution. Pure glycerol has considerably lower conductivity than water, since the sodium ions from pyranine do not fully dissociate in glycerol as they do in water. Thus, it is reasonable to assume that pyranine may have a negative charge due to partial dissociation of the sulfonate groups. At *f* = 10 mHz, the impedance modulus decreases from 3.4 to 2.4 MΩ upon illumination, while the phase is about −75° in both cases. At low frequencies (long time intervals), the graph suggests capacitive behavior with an increase in capacitance upon illumination.

As mentioned above, illumination increases the ion conductivity of the pyranine-doped glycerol. However, one should also note that the electrode records the photovoltage over longer periods (smaller frequencies), where the capacitive behavior plays a larger role. In the linearized Poisson–Boltzmann equation, electrode capacitance per area is given by *C* ≈ ε_0_ε_r_/λ, where ε_0_ is the permittivity of vacuum, ε_r_ is the relative permittivity, and λ is the Debye length that scales inversely with the square of the concentration of the charged species.^23^ This equation suggests that the capacitance can increase by an increase in either the permittivity or the charge concentration. We know from Figure 5a that relatively little charge (if charge is proportional to *V*_oc_) builds up during impedance measurements down to 10–100 mHz, since such measurements take significantly shorter time than the several hours needed to saturate the photovoltage in glycerol. It is also seen from the fluorescence spectra in Figure 1b) that pyranine is protonated in glycerol, and there is therefore no obvious source of negative charge due to laser excitation. This could point toward change in relative permittivity as a reason for the increase in capacitance.

A possible explanation of the photovoltage observed in glycerol is that the laser illumination causes local heating, migration of negative pyranine ions, and a photothermal voltage that grows to the order of 0.2 V. Supporting evidence for this is found by looking at other salts and dyes, which gave rise to changes in voltage of a few mV as discussed above. If a temperature difference between the electrodes is the sole cause, then one may expect voltages of the order of 1 mV per Kelvin.^24^ Thus, a few Kelvin increases in local temperature could possibly explain the results for NaCl and methyl red solutions described above. On the other hand, for 1 mM in pyranine glycerol, the voltage is of the order of 0.2 V and cannot be explained by a simple change in the Debye length as done in previous research.^24^ Moreover, if the photothermal effect was the only underlying reason for the voltage, one should expect different voltage kinetics in water and also not a large difference between methanol and glycerol, which both protonate pyranine. One therefore needs to search for other explanations. One possibility is that illumination of pyranine causes the excited state complex to be polarized such that the glycerol in the vicinity reacts by orienting its hydroxyl groups and thereby forming an effective negatively charged complex upon adsorption at the electrode surface, where the local permittivity ε_r_ and charge density may increase. For a concentration of 1 mM pyranine, these local complexes form at the electrode and in the bulk, where the latter will diffuse toward the electrode as governed by the viscosity of the solution. In Figure 7, the complexes have been assigned an effective negative charge in order to provide a simplified diagram of the diffusion kinetics.

Similar observations as for glycerol were made also for ethylene glycol, which is a diol that shares some properties with glycerol, in particular the ability of more than one hydroxyl group to reorient and react to the local environment. The ratio *I*_blue_/*I*_green_ in Figure 2b is smaller for ethylene glycol than glycerol for any molar fraction, which suggests that ethylene glycol has a smaller ability to protonate pyranine in the presence of water, possibly caused by fewer hydroxyl groups per molecule. The impedance spectrogram (not shown) exhibited almost no change in conductivity at larger frequencies, but a small and notable increase in capacitance was observed for small frequencies, the latter qualitatively similar to that found for glycerol. The photovoltage is seen in Figure 5a to increase to a slightly smaller value than that of glycerol, which might be related to fewer hydroxyl groups available, thus giving rise to less effective negative charge of the pyranine.

On the other hand, pyranine in methanol was only found to give rise to a negative photovoltage, as seen in Figure 5a. The voltage first decreases in methanol just as for water, before it starts to increase and decrease again until it apparently reaches a saturation value of about −40 mV. These fluctuations were found to vary slightly from experiment to experiment, but the polarity always remained the same. Although the initial increase in voltage is possibly driven by diffusion as for water, it is also clear that the subsequent fluctuations suggest a mechanism more complicated than proposed in Figure 7, possibly driven by local evaporative cooling since methanol has a higher evaporation rate than any other liquid used here. However, it appears reasonably clear that positive charge is responsible for the negative photovoltage, which is surprising since methanol protonates pyranine, as seen in Figure 1a. From Figure 1a, an isosbestic point at 482 nm is observed, which suggests that the stoichiometry does not change for different molar ratios. From Figure 2b, the ratio *I*_blue_/*I*_green_ is smaller for methanol than for ethylene glycol or glycerol for different molar fractions in the presence of water. This is not surprising, since methanol only has a single hydroxyl group and lower permittivity than both ethylene glycol and glycerol. Unlike glycerol and ethylene glycol, methanol has only one hydroxyl group, and this sometimes has significant effects. For example, methanol is known to prevent charge transfer less efficiently at interfaces than glycerol in the presence of water^25^ and is believed to form localized structures or percolating networks that separate it from the water hydrogen bond network.^26–28^ A possibility for the observed photovoltage for methanol is that the assumed pure methanol contains small amounts of water (from the air), which dissolves small amounts of pyranine without significantly changing the observed fluorescence emission. This may lead to a small photovoltage as seen in Figure 5a. Another possibility is that the excited-stated complex wherein methanol and pyranine interact gives rise to a polar complex, which orients itself and gives rise to a net-positive charge once it reaches the electrode. The experiments provided here cannot distinguish between the two possibilities, and further investigations are outside the scope of this work.

The findings in Figures 5 and 6 give reason to believe that for water, the initial increase in photovoltage toward −0.2 V is governed by diffusion of positive charge toward the electrode. Similarly, it is also proposed that the positive photovoltage in ethylene glycol and glycerol is governed by diffusion. In order to further investigate this possibility, the photovoltage in different water mixtures was investigated, and the results are displayed in Figure 8. Figure 8a shows the open-circuit voltage for glycerol–water molar ratios χ = 0 (green line), χ = 0.058 (orange line), χ = 0.11 (brown line), and χ = 1 (violet line). It is observed that for χ = 0.058 (orange line), the voltage decreases slower than in water and to a much smaller value. On the other hand, for χ = 0.11 (brown line), the voltage first becomes negative and decreases, before it switches polarity and becomes positive and finally saturates after less than two hundred seconds. This suggests that two charges of opposite sign are at work, and that these operate at different time scales.

**Figure 8:**
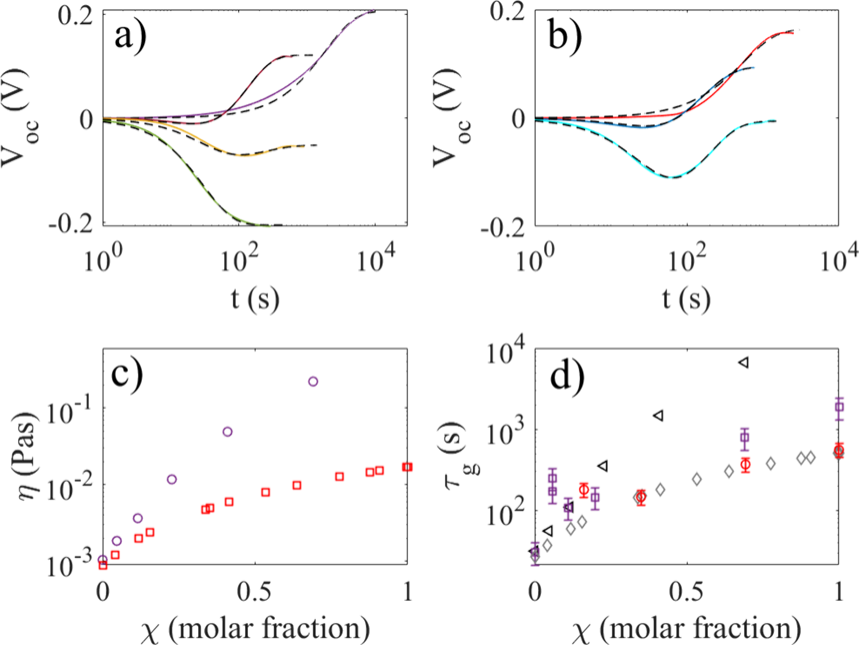
In panel (a), the open-circuit voltage versus time is displayed for glycerol–water molar ratios of χ = 0 (green line), χ = 0.058 (orange line), χ = 0.11 (brown line), and χ = 1 (violet line). In panel (b), the open-circuit voltage versus time is displayed for ethylene glycol–water molar fractions of χ = 0.16 (cyan line), χ = 0.35 (blue line), and χ = 1 (red line). The black, dashed lines in panels (a) and (b) are fits of eq 4 to the experimental data. In panel (c), the viscosity of glycerol–water mixtures (violet circles) and ethylene glycol–water mixtures (red squares) was extracted from the literature.^29,30^ In panel (d), the time constant τ_g_ obtained from fitting eq 4 is displayed as a function of ethylene glycol–water molar fractions (red circles) and glycerol–water molar fractions (violet squares). The calculated time constants using the known diffusion coefficient of pyranine in water^15^ together with the data for the viscosity from the literature^29,30^ are displayed as black triangles (glycerol–water) and gray diamonds (ethylene glycol–water) in panel (d); see the text for detailed explanation.

In Figure 8b, the photovoltages in ethylene glycol–water molar fractions χ = 0.16 (cyan line), χ = 0.35 (blue line), and χ = 1 (red line) are shown. In the case of χ = 0.16 (cyan line), the voltage first decreases to −0.1 V before increasing toward 0 V. Here, the negative and positive charges effectively cancel each other. For χ = 0.35 (blue line), one observes similar behavior as for glycerol–water fraction χ = 0.11, with an initial negative followed by a positive voltage. These observations suggest that also water/ethylene glycol mixtures are governed by two differently charged species operating at two different time scales. The time scale for the positively charged species is much smaller for ethylene glycol than for glycerol, which suggests that viscosity plays an important role for the latter. Figure 8c shows the viscosity of glycerol–water mixtures (violet circles) and ethylene glycol–water mixtures (red squares) as extracted from data in the literature.^29,30^

Here, it is proposed that the observations in Figure 8a,b can be explained if one assumes that charges of different polarity experience different diffusion constants governed by the viscosity. First, there are “free” water molecules that form hydrogen bonds in coordination with typically four other water molecules. Protons have a large diffusion coefficient of *D_v_* = 9 × 10^–9^ m^2^/s^20^, possibly due to hopping along hydrogen bonds between water molecules. Let *n_v_* be the density of protons at the electrode at any time, and in the stable state, it is *n*_0v_. This is contrasted with “bound” water that is fixed by the hydroxyl groups of polyols and therefore restrict water molecules and cause negatively charged pyranine complexes to have a diffusion coefficient *D_g_,*which depends on the viscosity of the mixture. It has been shown that in pure water, the translational diffusion coefficient for pyranine is 2.68 × 10^–10^ m^2^/s at a temperature of *T* = 295 K, whereas in more viscous environments, the coefficient is larger.^15^ The negative charges have density *n_g_* at the electrode, and *n*_0g_ in the stable state. For both species, one has *n_i_* (*t* = 0) = 0 and *n_i_* (*t*→∞) = *n*_0i_, where *i* = *v*,*g*. In each of the two channels, the ions diffuse independently of each other, which means that one can separate their contributions. Here, only the kinetics is of interest, and the diffusion equation may therefore be spatially homogenized to become

$$\frac{dn_{i}}{dt}\approx D_{i}\frac{n_{0i}-n_{i}}{L_{i}^{2}}$$ 1

where *L_i_* is the distance average ionic travel distance. Solving eq 1 gives

$$n_{i}(t)\approx n_{0i}(1-e^{-t/\tau_{i}}),\;\;\;\;\;\;\;\;\;\;\;\;\;\;\;\;\tau_{i}=\frac{L_{i}^{2}}{D_{i}}$$ 2

The change in potential is through Gauss’ law related to the number of ions as

$$V\approx \sum_{i}U_{i}n_{i}$$ 3

Here, *U*_*i*_ = *q*_*i*,eff_/(*A*ε_0_ε_r_), where ε_r_ is the relative permittivity of the liquid, ε_0_ is the permittivity of vacuum, *q_i_* is the effective charge of the diffusing charges, and *A* is the electrode area. Equations 2 and ([Disp-formula eq3]) give

$$V_{\mathrm{oc}}(t)=V_{v}(1-e^{-t/\tau_{v}})+V_{g}(1-e^{-t/\tau_{g}})$$ 4

where *V*_*i*_ = *n*_0i_ *q_i_A*/(ε_0_ε_r_). Here, *V_i_* will be treated as fitting constants since eqs 1–4 are based on spatial homogenization and therefore do not capture the constants well. It should be emphasized that eq 4 assumes two different charged species that exhibit different time constants. It neglects any secondary diffusion effects. For example, the increase in photovoltage from −0.2 to −0.07 V in Figure 4b might be such a secondary effect, which is not accounted for in eq 4. However, it is also noted that the photovoltage remains near the peak of −0.2 V for several hundred seconds in Figure 4b, which is significantly longer than the time scales required for the photovoltages to flip sign in Figure 8a,b. This may suggest that it is the first diffusion from the bulk toward the electrode that plays the main role in Figure 8a,b, thereby supporting the hypothesis adopted here. One factor that could also contribute is polarity, through the relative permittivity of the liquid. As seen in Figure 6b, the increase in capacitance may be due to a change in permittivity ε_r_. If that is the case and the main change in photovoltage given by eq 3 is due to changes in permittivity and not charge concentration, one must expect the permittivity to exhibit an exponential change to be able to derive an equation similar to eq 4 from eq 3. Currently, there is not enough evidence pointing toward such a possibility to justify further pursuit, although it should not be ruled out entirely with the given experimental data.

The black dashed lines in Figure 8a,b show fits of eq 4 to the experimental data, with the different fitted constants given in Table 1. We note from Table 1 that the magnitude of *V_v_* decreases toward zero with increasing glycerol–water or ethylene glycol–water fraction. This suggests that the positive charges responsible for *V_v_* decrease as more glycerol and ethylene glycol molecules are added.

**Table 1:** Fitting Parameters Obtained from Fitting eq. 4 to the Experimental Data

	χ	τ_v_ (s)	τ_g_ (s)	V_v_ (mV)	V_g_ (mV)
glycerol–water mixtures	0	30		–205	0
	0.058	30	249	–84	32
	0.11	30	109	–98	219
	0.20	30	144	–63	235
	0.69	30	787	18	149
	1.0		1866	0	205
ethylene glycol-water mixtures	0.16	30	180	–187	181
	0.35	30	146	–72	166
	0.69	30	369	–7	115
	1.0		557	0	163

We also note that for pure water, one has τ_v_ = 30 s, which corresponds to *L* = 0.5 mm using *D_v_* = 9 × 10^–9^ m^2^/s for protons and τ_v_ = *L_v_* ^2^/*D_v_*. For comparison, pyranine ions diffuse about 0.09 mm in 30 s in pure water. According to absorbance measurements like those in Figure 1a, the absorbance of 1 mM pyranine is 3.9 × 10^3^ m^–1^, and Beer–Lamberts law then states that the laser intensity has been reduced to 14% of its initial value after propagating *L_v_* = 0.5 mm into the solution. After propagating 1 mm, i.e., one-fifth of the length between the ITO electrodes, only 2% of the laser intensity has not been absorbed. The value *L_v_* = 0.5 mm therefore represents a reasonable effective distance over which the protons need to travel to reach the ITO electrode.

In Figure 8a,b, good fits of eq. 4 to the experimental data could be obtained keeping τ_v_ constant and equal to the value found for pure water, while τ_g_ was allowed to vary as seen in Table 1. Figure 8d shows the time constant τ_g_ obtained from fitting eq. 4 for ethylene glycol–water mixtures (red circles) and glycerol–water mixtures (violet squares). It is noted that τ_g_ is significantly smaller in the presence of ethylene glycol–water mixtures than in glycerol–water mixtures, in particular at higher molar fractions. In both cases, the time constants increase with molar fraction, which can be attributed to an increase in viscosity. The increase in τ_g_ with molar fraction can be explained by considering the Einstein relationship *D_g_* = *k*_B_ *T*/6πηr, where *k_B_* = 1.38 × 10^–23^ J/K, *r* is the hydrated molecular radius and η the viscosity as determined from the data^29,30^ presented in Figure 8c. A simple estimate of the radius of pyranine based on its molecular mass (524 g/mol) gives *r* ≈ 5 × 10^–10^ m, while an estimate using the Einstein relationship and a diffusion coefficient *D_p_* ≈ 2.68 × 10^–10^ m^2^/s^15^ in pure water of viscosity η_v_ = 10^–3^ Pas results in a hydrated radius of *r* ≈ 8 × 10^–10^ m^15^. Although this hydrated radius is likely to change with an increasing molar fraction, it will here be assumed to be constant.

If only the viscosity in the Einstein relationship changes when adding glycerol to water, then the diffusion coefficient of pyranine in glycerol–water mixtures is *D_g_* = (η_v_/η_g_)*D_p_*. The time constant is given as τ_g_ = *L_g_* ^2^/*D_g_*. The value of *L_g_* is not known, but somewhere in the range of 0.09 to 0.5 mm appears reasonable. However, the available data cannot tell whether a particular choice of *L_g_* is significant. A simple possible approach is to extrapolate from pure water, i.e., assume that in pure water, the negative pyranine ions perform Brownian motion over a time interval τ_v_ = 30 s with a diffusion coefficient *D_p_*. Then, one can obtain an estimate of the expected time constant by writing τ_g_ = (η_g_/η_v_)­τ_v_. Using viscosity data from the literature^29,30^ to obtain η_g_ in the equation τ_g_ = (η_g_/η_v_)­τ_v_ results in the black triangles for glycerol/water mixtures and gray diamonds for ethylene glycol–water mixtures in Figure 8d. Except for the lower molar fractions, the agreement between the data and simulations for ethylene glycol is very good. For glycerol, there are larger deviations, but still a qualitative agreement with the fitted parameters in Table 1. At higher glycerol fractions, the deviations between the black circles and the blue squares in Figure 8 d are significant, for reasons not known. While there might be a reduction in hydration radius as the glycerol molecules surround the pyranine ions, the estimates above suggest that a bare ion can only reduce the radius and therefore the time constant by a factor of 0.6 (5/8).

It should be mentioned that the fits presented in Table 1 are based on the assumption that time constant τ_v_ remains constant and equal to that of water. On the other hand, the value of τ_g_ is allowed to vary with the viscosity of the solution. This assumes that the H^+^ ions move in the hydrogen bonding network formed by water molecules, while the pyranine complex giving rise to the negative charge experiences the interaction with the alcohol and therefore has to move through a more viscous environment. The negative charge could be due to partial dissociation of the sulfonate groups, which are heated upon laser illumination and cause a positive photothermal voltage. However, such an explanation is not well supported by the findings for water and all alcohols as described in connection with Figures 4 and 5. Another possible explanation is light-induced effective negative charge on the pyranine complex, causing a positive photovoltage, but in this case, the nature of the molecular complex is not well understood.

While the theoretical background for the negative charge is not well understood and its exact origin cannot be determined from the experimental data presented here, the photovoltage for both the positive and negative charge appears to be well modeled by eq. 4. The choice of a fixed τ_v_ and a variable τ_g_ is a model choice, which appears to be at least partially supported by the good agreement between eq 4 and the experimental data in Figure 8a,b, in addition to the fitted and theoretical values of τ_g_ in Figure 8d. However, it should also be pointed out that it is possible to fit eq 4 to the experimental data while varying both τ_v_ and τ_g_. This will result in only marginally better fits since the experimental data exhibit some deviations from the exponential shape and still give rise to one small (close to τ_v_ = 30 s) and one large variable time constant scaling with viscosity consistent with the interpretation given above. The fact that the suggested mechanism applies to at least two different alcohol–water mixtures suggests that it merits further scrutiny. In particular, it would be of interest to probe the molecular diffusion in the microscopic environment with and without illumination, but this is outside the scope of the current work.

## 4 Conclusions

In the current work, it is demonstrated that the polarity of the photovoltage generated by the photoacid pyranine depends on the glycerol to water weight fraction. While illumination of the photoacid in water releases positive H^+^ ions, illumination in glycerol or ethylene glycol results in negative charges that use a much longer time to reach the electrode with a time constant depending on the molar fraction in water. A diffusion model is proposed, wherein the two oppositely charged species are given different diffusion constants. The two oppositely charged species give rise to widely different kinetics, such that the photovoltage may become negative before it switches and becomes positive for a certain range molar fractions, but remains monopolar outside this range.

These findings may have further use when investigating photoacidity in liquid mixtures or composite materials with potential applications in light energy-harvesting. If one can design different diffusion channels wherein ions of opposite polarity are released, then it should be possible to obtain both controllable polarity and kinetics simultaneously. If more similar diffusion coefficients can be found for positive and negative ions, this may also pave the way for new types of pn-junctions.

## References

[1] Chakraborty R., Berglund K. A. (1992). Steady state fluorescence spectroscopy of pyranine
as a trace extrinsic probe to study structure in aqueous sugar solutions. J. Cryst. Growth.

[2] Gonzales J., Sakaue H. (2022). Novel optical temperature and phase
change sensor based
on the response of hydroxypyrene to sucrose in water ice. Sensors and Actuators A.

[3] Kataoka S., Harada M., Okada T. (2021). Microscale pH inhomogeneity
in frozen
NaCl solutions. Phys. Chem. Chem. Phys..

[4] Zhao Z., Zhang F., Zhang Z. (2018). A facile fluorescent “turn-off”
method for sensing paraquat based on pyranine-paraquat interaction. Spectrochimica Acta A: Molecular and biomolecular spectroscopy.

[5] Saha T., Sengupta A., Hazra P., Talukdar P. (2014). In vitro sensing of
Cu+ through a green fluorescence rise of pyranine. Photochem. Photobiol. Sci..

[6] Xue P., Ding J., Jin M., Lu R. (2017). Rapid gel-to-sol transition
triggered by a photoacid generator under low-power light. J. Mater. Chem. C.

[7] White W., Sanborn C. D., Reiter R. R., Fabian D. M., Ardo S. (2017). Observation
of photovoltaic action from photoacid-modified Nafion due to light-driven
ion transport. J. Am. Chem. Soc..

[8] Bae J., Lim H., Ahn J., Kim Y. H., Kim M. S., Kim I. (2022). Photoenergy
harvesting by photoacid solution. Adv. Mater..

[9] Yucknovsky A., Shlosberg Y., Adir N., Amdursky N. (2023). Photocurrent generation
and polarity switching in electrochemical cells through light-induced
excited state proton transfer of photoacids and photobases. Angew. Chem., Int. Ed..

[10] Dai X., Berton C., Kim D. J., Pezzato C. (2024). Wiring proton gradients
for energy conversion. Chem. Sci..

[11] Dai X., Gangadharappa C., Chen S., Cherif S. E., Gericke C., Yadav B., Pezzato C., Kim D. J. (2025). Non-equilibrium
proton gradients for photoenergy conversion. ACS Appl. Energy Mater..

[12] Wu L., Qi J., Zhang L., Yu L., Gao H., Gao J., Ju J., Yao X. (2024). Self-powered photoelectric sensors based on hydrogel
diodes doped with photoacids. Chem. Eng. J..

[13] Patel P. K., Foguel M. V., Calvo-Marzal P., Chumbimuni-Torres K. Y. (2021). Reversible
photovoltage generation using metastable-state photoacids in the solid
state with visible light. J. Phys. Chem. C.

[14] Nandi R., Amdursky N. (2022). The dual use of the pyranine (HPTS)
fluorescent probe:
A ground state pH indicator and an excited-state proton transfer probe. Acc. Chem. Res..

[15] Chakraborty S., Nandi S., Bhattacharyya K., Mukherjee S. (2019). Time evolution
of local pH around a photo-acid in water and a polymer hydrogel: Time
resolved fluorescence spectroscopy. ChemPhysChem.

[16] Helseth L. E. (2012). Pyranine-induced
self-assembly of colloidal structures using poly­(allylamine-hydrochloride). J. Colloid Interface Sci..

[17] Barrash-Shiftan N., Brauer B., Pines E. (1998). Solvent dependence
of pyranine fluorescence
and UV-visible absorption spectra. J. Phys.
Org. Chem..

[18] Mohammed O. F., Dreyer J., Magnes B. Z., Pines E., Nibbering E. T. J. (2005). Solvent-dependent
photoacidity state of pyranine monitored by transient mid-infrared
spectroscopy. ChemPhysChem.

[19] Quine Z., Goun A., Laforge F., Rabitz H., Law C. K. (2023). Chemically
sensitive fluorescence imaging of colliding microdroplets. Phys. Fluids.

[20] Haghighat S., Ostresh S., Dawlaty J. M. (2016). Controlling
proton conductivity with
light: A scheme based on photoacid doping of materials. J. Phys. Chem. B.

[21] Nagarkar S. S., Horike S., Itakura T., Le Ouay B., Demessence A., Tsujimoto M., Kitagawa S. (2017). Enhanced and optically
switchable
proton conductivity in a melting coordination polymer crystal. Angew. Chem..

[22] Glancy J., Luo S., Kim T. Y., Ardo S. (2022). Identification of photoacidic behavior
using AC and open-circuit photoelectrochemical techniques. ECS J. Solid State Sci. Technol..

[23] R. J., Hunter “Introduction to modern colloid science”, Oxford Science Publications, 1 ^st^ ed., 1993.

[24] Wang Y., Jia K., Zhang S., Kim H. J., Bai Y., Hayward R. C., Suo Z. (2022). Temperature
sensing using junctions between mobile ions and mobile
electrons. Proc. Natl. Acad. Sci. U.S.A..

[25] Helseth L. E. (2024). Charge
transfer quenching and maximum of a liquid-air contact line moving
over a hydrophobic surface. Langmuir.

[26] Guo J. H., Luo Y., Augustsson A., Kashtanov S., Rubensson J.-E., Shuh D. K., Ågren H., Nordgren J. (2003). Molecular structure
of alcohol-water mixtures. Phys. Rev. Lett..

[27] Dougan L., Bates S. P., Hargreaves R., Crain J., Finney J. L., Reat V., Soper A. K., Fox J. P. (2004). Methanol-water solutions:
a bi-percolating liquid mixture. J. Chem. Phys..

[28] Bako I., Megyes T., Balint S., Grosz T., Chihaia V. (2008). Water-methanol
mixtures: topology of hydrogen bonded network. Phys. Chem. Chem. Phys..

[29] Takamura K., Fischer H., Morrow N. R. (2012). Physical properties of aqueous glycerol
solutions. J. Petroleum Sci. Eng..

[30] Hayduk W., Malik V. K. (1971). Density, viscosity,
and carbon dioxide solutibility
and diffusivity in aqueous ethylene glycol solutions. J. Chem. Eng. Data.

